# The Effect of Habitual Speech Rate on Speaker-Specific Processing in English Stop Voicing Perception

**DOI:** 10.1177/00238309231188078

**Published:** 2023-08-09

**Authors:** Connie Ting, Yoonjung Kang

**Affiliations:** Department of Linguistics, University of Toronto, Canada; Department of Linguistics, McGill University, Canada; Department of Linguistics, University of Toronto, Canada; Department of Language Studies, University of Toronto Scarborough, Canada

**Keywords:** Speech rate, VOT, individual speaker variation, speech perception

## Abstract

This study investigates listeners’ ability to track individual speakers’ habitual speech rate in a dialogue and adjust their perception of durational contrasts. Previous studies that found such adjustments are inconclusive as adjustments can be attributed to exemplars of target structures in the dialogue rather than perceptual calibration of habitual speech rates. In this study, English listeners were presented with a dialogue between a fast and slow speaker, containing no stressed syllable-initial voiceless stops. Listeners then categorized /pi/-/bi/ syllables differing along a voice onset time continuum. Results did not show conclusive evidence that listeners’ response differed systematically depending on speakers’ habitual speech rate.

## 1 Introduction

In everyday communication, listeners deal with highly variable speech. One source of variation is changes in speaking rate. Languages make use of durational differences to signal phonological contrasts. In English, for example, voice onset time (VOT), the interval between the release of a stop and onset of vocal cord vibration, is a durational contrast that differentiates voiced from voiceless stop phonemes. Speech rate effects have been found for stop voicing contrasts such that, as speech rate gets faster, VOT for voiceless stops shortens (Kang et al., 2018; Miller et al., 1986; Miller & Volaitis, 1989). This can change the boundary between voiced and voiceless stops (Sawusch & Newman, 2000; Summerfield, 1981) and create an (increase in) overlap between voiced and voiceless stops in fast speech (Kessinger & Blumstein, 1997). This poses a potential problem for listeners because a speaker’s realization of /b/ in slower speech may have similar VOT values as a speaker’s realization of /p/ in faster speech.

Research has shown that listeners are sensitive to contextual variations such as speech rate and are able to compensate for this variation by tuning their perception of VOT relative to the given speech rate (Kidd, 1989; Miller, 1987; Miller & Dexter, 1988; Miller & Liberman, 1979; Sawusch & Newman, 2000). As VOT increases as speech rate slows, the same VOT was more often perceived as /b/ in slow speech, but as /p/ in fast speech. Similar speech rate effects have been found in the perception of vowel duration (Reinisch, 2016; Reinisch & Sjerps, 2013). Researchers have also examined whether listeners are able to track the speech rate of speakers over an extended period of time (habitual or global rate), rather than the speech rate of a carrier sentence directly preceding a target word (local rate) (Baese-Berk et al., 2014; Maslowski et al., 2019b). Results showed that listeners tracked variation in the overall speech rate of individual speakers over an extended period of time and that their knowledge of the speakers’ habitual speech rate influenced speech perception.

Much of this research has probed listeners’ perception of lab speech and speaker utterances presented as non-conversational speech. However, less is known about the perception of durational cues in other contexts, such as spontaneous speech and conversational dialogues, in which influences of speech rate on VOT may be variable. For example, previous work on corpus data has shown that different measures of speakers’ speech rate can lead to different results regarding a correlation between speech rate and VOT (Stuart-Smith et al., 2015). It has also been argued that optimal category boundaries can be obtained independently of speech rate (Nakai & Scobbie, 2016). Another way in which we can probe speech rate effects in its more usual habitat is through conversational speech. In daily life, listeners are often faced with situations in which multiple people are involved in a shared dialogue. To the best of our knowledge, Reinisch (2016) is the first to examine this type of speech context. As each speaker provides unique speech rate information, the process of rate normalization in this context relies on the listeners’ ability to track individual speakers separately and make use of their knowledge of speaker-specific properties in perception. Previous research suggests that listeners are able to track duration properties in a speaker-specific fashion (Allen et al., 2003). Such evidence suggests that listeners likely also track speaker-specific rate information to facilitate speech perception.

Reinisch (2016) sought to extend these findings by testing speaker-specific effects of speech rate on listeners’ vowel length perception in German. The study examined speech rate effects in the context of a conversation between two speakers. Listeners heard a 2-min dialogue between two female native speakers of German, varying in rate (fast vs. slow) and order (first vs. second). Following the dialogue, listeners completed a phonetic identification task in which they heard words of minimal pair continua differing in the /a/-/a:/ duration contrast and were asked to identify the word they heard. Results showed that listeners retained speech rate information, resulting in a shift in perception of the vowel contrast depending on the speech rate of each speaker; that is, there were more /a:/ responses for the faster speaker than the slower speaker.

These results are restricted to the context of vowel duration contrasts, and it remains to be seen how generalizable this finding is to other types of duration contrasts, specifically to consonantal contrasts like VOT. There are reasons to suspect that habitual speech rate may have different effects on the perception of vowels and consonants. It has been shown that changes in duration due to speech rate are reflected primarily in vowels rather than consonants (Gay, 1978; Port, 1981). This asymmetry may bias listeners to attend more to vowels than consonants in tracking speech rate leading to a more reliable habitual speech rate effect on the perception of vowels than consonants. Another difference between consonants and vowels is that vowels are ubiquitous in speech, such that any amount of exposure to a speaker’s speech rate provides ample tokens of vowels to be used for comparison in later perception. Moreover, Reinisch’s (2016) exposure dialogue contained 23 tokens of /a/ and 15 tokens of /a:/ in a 2-min dialogue. Thus, it remains ambiguous whether the effect of habitual rate found by Reinisch (2016) is indeed evidence for the use of general information about speech rate or whether it is the result of durational properties of the vowel tokens contained in the exposure. This study aims to provide a more stringent test of speakers’ habitual rate on subsequent speech perception using a consonantal contrast. The results of three experiments will be discussed in the subsequent sections. Experiment 1 replicates the study by Reinisch (2016), using VOT. To preview findings, no clear habitual speech rate effect was found.

One possible explanation for the lack of speech rate effect is that listeners were unable to track speakers’ habitual speech rate due to difficulty distinguishing between speakers of the same gender and age. Another explanation is that listeners aggregated speech rate information from the two speakers in the dialogue. This led to two follow-up experiments testing whether the lack of speech rate effect would persist when listeners heard speakers of different genders (Experiment 2) or when speakers’ habitual speech rates were comparable but varied across conversations (Experiment 3).

## 2 Method

### 2.1 Participants

A total of 608 listeners were recruited online and paid for their participation using Amazon Mechanical Turk (MTurk). Data were retained for 584 participants (196, 186, and 202 participants in Experiments 1, 2, and 3, respectively) who were self-identified native speakers of North American English and reported normal speech, hearing, and vision.

### 2.2 Stimuli

A 324-word dialogue between two speakers was scripted such that no stressed syllable-initial voiceless stops were included. Voiced stops were not avoided on the basis that voiceless stops have been reported to be more affected by speech rate compared with their voiced counterparts (Miller et al., 1986). It is worth noting that voiceless stops in other contexts (e.g., word medial and unstressed) were not completely absent from the dialogue. In other words, voiceless stops were strictly avoided only in contexts where aspiration is expected. Creating a natural dialogue without any instances of stops proved practically impossible. We consider the effects of the distribution of voiced and voiceless stops in the dialogue in Section 4.

Four voice actors (two male and two female) were recruited from https://www.fiverr.com/, and they were all self-identified native speakers of North American English. The speakers recorded both roles of the dialogue and were instructed to read the dialogue at a comfortable rate. The dialogue recordings were segmented at phrase boundaries and labeled according to the speaker-turn (A or B). Phrase durations were measured to determine the natural speech rate for each speaker. The mean sentence durations of the four speakers were 4.487, 4.306, 5.100, and 5.556 s for F1, F2, M1, and M2, respectively.

The four speakers were matched to create two same-gender pairs for Experiments 1 and 3 and two different-gender pairs for Experiment 2. Phrase durations were then manipulated to create two speech rate conditions (fast and slow), such that, for each pair, the fast version was compressed to be 15% shorter, and the slow version was expanded to be 10% longer than the average of the two speakers’ natural speech rate. Manipulated phrases were spliced back together leaving 250 ms of silence between utterances. The amount of rate change and inter-utterance gap were chosen to reflect that of the study by Reinisch (2016) while ensuring that the resulting dialogue was natural whereas the speech rate was distinct enough to be recognized as fast or slow. For each speaker pair, four versions of the dialogue were created such that each speaker was heard at each speech rate (fast and slow) and in each role (A and B). The different combinations of speaker pairs and speech rate condition in each of the three experiments are summarized in Table 1.

**Table 1.** Summary of Combinations of Speaker Pair and Speech Rate Condition in Each of the Three Experiments.

Experiment	Speech rate condition	Speaker pairs
		M = Male; *F* = Female
1 (same gender/different rate)	fast-slow/slow-fast	M1-M2/M2-M1
		F1-F2/F2-F1
2 (different gender/different rate)	fast-slow/slow-fast	F1-M1/M1-F1
		F2-M2/M2-F2
3 (same gender/same rate)	fast-fast/slow-slow	M1-M2/M2-M1
		F1-F2/F2-F1

Each speaker also recorded 10 repetitions of the words “bee” /bi/ and “pee” /pi/. One representative token of each word was chosen as the base token for creating the stimuli for the identification task. For each speaker, stimuli were made by splicing the aspiration of the speaker’s /pi/ token onto the vowel of the speaker’s /bi/ token. The VOT duration was then manipulated to create a VOT continuum ranging from 0 to 50 ms in 11 equal steps of 5 ms. For each speaker within a pair, the vowel duration was kept constant at the average vowel duration within the pair. All stimuli were manipulated using *Praat’s* PSOLA algorithm (Boersma & Weenink, 2018).

### 2.3 Procedure

The experiments were built using *jsPsych* (De Leeuw, 2015; Ryu, 2018) and completed by participants through the MTurk online system. Participants were instructed to make sure they were wearing headphones and were asked to provide the model of the headphones. They also completed a short demographic and language background questionnaire before the experiment. Participants were randomly assigned to one of the three experiments. Within each experiment, participants heard one of four versions of the dialogue (2 roles × 2 rates) for one speaker pair. Once the dialogue was finished, participants completed a self-paced identification task in which they listened to the speakers say either “pee” or “bee” and clicked the corresponding word on the screen to indicate which word they heard. The button on the left side of the screen was always “pee.” After each response, the next stimulus would play after 500 ms. The two speakers’ word items were presented intermixed. Each stimulus was repeated three times, resulting in a total of 66 trials for each listener (11 VOT steps × 2 speakers × 3 repetitions). Stimuli were randomized for each participant with the restriction that no identical token be presented twice in a row.

After the identification task, participants answered a multiple-choice question. The relevant information appeared within the last sentences of the dialogue and so was used as a method to exclude participants who were not paying attention during the experiment. Those who answered incorrectly were excluded from the analysis (*n* = 69), and data from the remaining participants were analyzed. The rate of this exclusion was comparable across the three experimental conditions (χ² = 1.317, df = 2, *p* = .5176). The experiment took approximately 5 min to complete.

If listeners are able to keep track of individual speakers’ habitual rate, we expect listeners to give more /p/ responses for the fast speech rate condition, compared with the slow condition. That is, if a speaker has a fast speech rate, a given VOT value will seem long relative to the speakers’ habitual rate, and therefore elicit more /p/ responses, and vice versa.

## 3 Results

**Figure 1.**
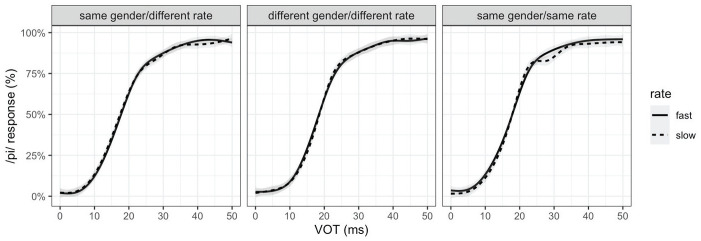
Proportion of /p/ responses over the VOT continuum for fast (solid line) versus slow (dashed line) speech rate in the dialogue, aggregated over speakers, for experimental conditions (Exp1: same gender/different rate, Exp2: different gender/rate, and Exp3: same gender/rate). The lines show loess smooths of the empirical data.

To ensure that the rate effect would not be affected by any lack of or weak VOT effect, participants whose perception did not show a significant effect of VOT (*p* > .1) in the expected direction for both speakers they heard were also excluded from further analysis (*n* = 67). In other words, participants who showed a trend in VOT effect in the right direction but failed to reach statistical significance for only one of the two speakers were excluded. The final analysis included data from 448 listeners (156, 142, and 150 participants from Experiments 1, 2, and 3, respectively). The exclusions based on the VOT effect also did not differ across experimental conditions (χ² = 2.0744, df = 2, *p* = .3544). Figure 1 shows the proportion of /p/ responses over the VOT continuum for the fast versus slow rate across the three experimental conditions. If speech rate had a systematic effect, we would see a higher /p/ response proportion for the fast rate (solid line) than the slow rate (dotted line). There is no systematic difference by speech rate, regardless of the conversational partner’s gender or speech rate.

**Figure 2.**
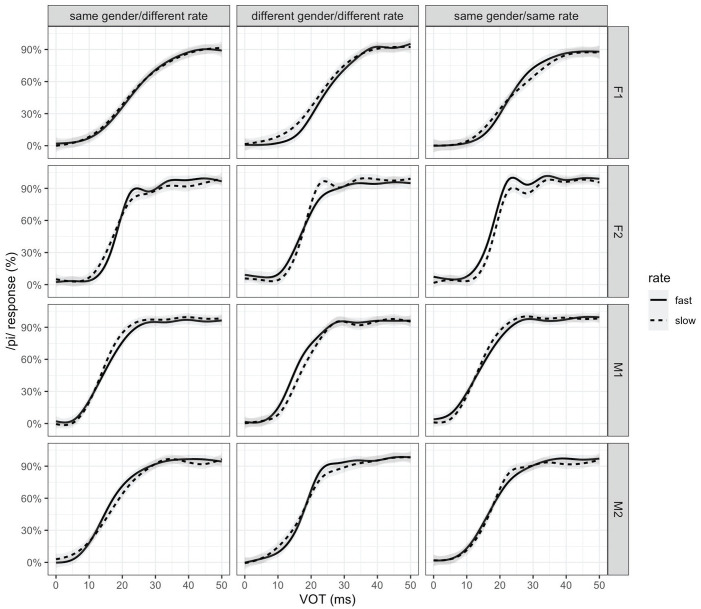
Proportion of /p/ responses over the VOT continuum for the fast (solid line) versus slow (dashed line) speech rate in the dialogue by speaker, for each experimental condition (Exp1: same gender/different rate, Exp2: different gender and rate, and Exp3: same gender and rate). The lines show loess smooths of the empirical data.

In Experiments 1 and 2, which involved speakers speaking at different rates within the dialogue, the speech rate effect is examined within participants. Experiment 3 involved speakers speaking at the same rate within the dialogue, with rate varying across conditions (i.e., half of the listeners heard a dialogue in which both speakers had a fast speech rate, the other half heard a dialogue in which both speakers had a slow speech rate). Thus, the speech rate effect in Experiment 3 is examined across participants. Note also that each participant heard only one speaker pair and rate combination. Figure 2 shows the results for each individual speaker across the experimental conditions.

Statistical analyses were conducted in *R* (R Development Core Team, 2021), and the *glmer* function of the *lme4* package (Bates et al., 2015) with a *bobyqa* optimizer was used. A logistic mixed-effects model was fit for each experiment, with response (/p/ coded as 1, /b/ coded as 0) as a dependent variable and VOT (ms; centered), Speaker (F1, F2, M1, M2; sum-coded), Rate (fast, slow; sum-coded), and the interaction of Rate with VOT and Speaker, as fixed factors. The random effects included by-Participant random intercepts and by-Participant random slope adjustments to VOT.

All three final models showed a significant effect of VOT (Exp1: β = 0.274, *z* = 23.6, *p* < .001; Exp2: β = 0.278, *z* = 22.5, *p* < .001; Exp3: β = .272, *z* = 23.1, *p* < .001), with more /p/ responses as VOT duration increased, as expected. We also found significant main effects of Speaker, with more overall /p/ responses for some speakers than others. There was no significant main effect of Rate (Exp1: β = −0.021, *z* = −0.273, *p* = .785; Exp2: β = 0.060, *z* = 0.748, *p* = .454; Exp3: β = −0.054, *z* = −0.213, *p* = .831), and no significant interaction of Rate and VOT, indicating the lack of RATE effect is consistent across the VOT range. The interaction of Rate and Speaker was only significant in Experiment 3, but a post hoc test with the Bonferroni adjustment shows no significant rate effect for any of the speakers. In short, the speakers’ speech rate in the dialogue had no systematic effect on stop perception. This finding is in contrast to the speech rate effects found in the study on German vowels that this study closely matched in design, with differences in the number of words and repetitions, but with about 10 times the number of participants (Reinisch, 2016). As our main finding is a null result, we conducted a post hoc power analysis of Experiment 1, using an estimated effect size of β = −0.25 of speech rate based on Reinisch’s Experiment 1. A power analysis using the *PowerSim* function of the *simr* package (Green & MacLeod, 2016) based on likelihood ratio tests of 1,000 simulations indicates that our Experiment 1 is sufficiently powered with 87.4% chance of detecting the effect (95% CI [85.18%, 89.39%]). Finally, a post hoc analysis revealed a significant interaction of Rate and Item Order for Experiment 1 (β = 0.347, *z* = 2.394, *p* = .017), reflecting a marginal effect of speech rate (*p* < .1) in the first half of the identification task in the expected direction and a marginal effect of speech rate (*p* < .1) in the second half of the identification task in the opposite direction. The same interaction is found when the Item Order is entered as a continuous variable (centered; β = 0.009, *z* = 2.466, *p* = .014). We review the implications of these results in Section 4.

## 4 Discussion

This study tested whether and how listeners keep track of individual speakers’ habitual rate in a short dialogue and make use of this information in a subsequent speech perception task. In all three experiments, we found no clear effect of habitual speech rate given the rate manipulation used in this study, which suggests that either listeners did not track individual speakers’ habitual rate, or they did track individuals’ speech rate, but the information did not affect their perception of the English /p/-/b/ contrast. These results are inconsistent with previous findings and warrant further discussion.

In this study, listeners heard /pi/-/bi/ syllables in which the stimuli varied in VOT duration. Crucially, however, these stimuli contained a vowel, which was kept constant in duration across the two speakers. Note that the duration of the vowel following the target stop provides listeners with local rate information (Sawusch & Newman, 2000) or itself serves as a secondary cue for the stop voicing contrast (Theodore et al., 2009). Here we use *local* rate information to refer to the adjacent context of the target word (i.e., the vowel), rather than an adjacent sentence which is also described in the literature as local (Maslowski et al., 2019b; Reinisch, 2016). It has been suggested that information more local (i.e., closer) to the target affects phonetic categorization more strongly than habitual rate information (i.e., rate information over an extended period of time), as normalization for local rate information occurs too early during speech perception for it to be influenced by habitual rate information (Maslowski et al., 2019a, 2020; Newman & Sawusch, 2009). Put differently, rate information plays a greater role in rate normalization the closer it is to the target. If listeners primarily made use of the invariable local rate information, it is then unsurprising that a clear rate effect was not found. However, previous studies found that the speech rate of preceding sentential context of a target stop reliably modulates VOT perception independent of the duration of the post-stop vowel (Kang et al., 2018; Toscano & McMurray, 2012, 2015). Therefore, the lack of habitual speech rate effect in our study cannot be attributed to the absolute dominance of the post-stop vowel length cue over preceding contextual speech rate cues in VOT perception.

Another possibility is that the rate manipulation was not large enough to observe a rate effect for the stop contrast. Previous work has shown that rate effects can be hard to detect (Shinn et al., 1985; Utman et al., 2000) and are fairly small when present (Toscano & McMurray, 2012). One way to address this is to perform a power analysis based on the effect size reported in previous studies. This was done based on the effect size found in Reinisch (2016), of which this study aimed to replicate. However, given that there exist inconsistencies in finding speech rate effects in previous work, and as we are focusing on a different kind of contrast, it is possible that a rate effect would emerge with larger rate manipulations in the dialogue context. Future studies involving different, more exaggerated, rate manipulations will be able to provide more context with regard to whether a rate effect can be found using a dialogue context more generally.

It could also be that listeners did not make use of the habitual speech rate of the speakers in VOT perception due to high variability in how speech rate affects VOT realization in English (Allen et al., 2003). English speakers differ widely in how much they modify their VOT production and perception both as a function of speech rate (Allen et al., 2003). Also, as speakers age, speech rate slows down, and while vowels are produced longer, VOT values become shorter, counter to the expected speech rate and VOT correlation (Benjamin, 1982). Given this interspeaker variability, a faster speech rate of one speaker is not a reliable indicator that they will produce a short VOT compared with a slower speaker. Moreover, the English VOT contrast may be robust enough against speaker-specific speech rate variation and a rate-independent VOT boundary may be effective enough for English voicing categorization in conversational speech, obviating the need for rate normalization (Heffner et al., 2017; Nakai & Scobbie, 2016).

The number of trials in the identification task may have led listeners to unlearn the habitual rate information gathered from the exposure dialogue. In other words, it could be the case that listeners did indeed make use of the habitual rate information early on in the identification task in the expected manner, but continued to keep track of speakers’ rate information as the task progressed which eventually diminished the effect of habitual speech rate from the earlier exposure dialogue. A post hoc analysis which showed a marginal effect of speech rate in the first half of the identification task for Experiment 1 supports this interpretation. However, if an effect of unlearning stems from the number of trials alone, these results are somewhat surprising given the fact that there was a higher number of trials in Reinisch’s (2016) study (224 trials per participant compared with 66 trials per participant in this study), yet a robust speech rate effect was still found. Other contributing factors could be the number of word pairs used for the identification task and the restriction of the stimuli continuum within each word pair. For example, Reinisch’s (2016) study included four word pairs, whereas this study included only one, which may have contributed to making the overall task less natural. It is also important to note that there was a marginal effect of speech rate in the second half of the identification task for Experiment 1 but in the opposite from expected direction. Furthermore, no order effect was found for Experiments 2 and 3. The extent to which these differences in stimuli and experiment conditions can account for the diverging results is a question we must leave for future work.

Finally, recall that Reinisch’s (2016) exposure dialogue contained a large number of target vowels, which were themselves varied in duration as part of habitual speech rate manipulation. It is thus possible that the speech rate effect found in Reinisch’s study was due to the speaker-specific vowel exemplars guiding the listeners’ categorization, rather than the speaker-specific habitual speech rate. Note that, in this study, the dialogue was designed to exclude instances of aspirated stops in stressed position to avoid instances where VOT duration would have covaried with speech rate. In the absence of matching exemplars, no speech rate effects on stop perception was found. At the same time, it is still possible that the speakers did track speaker-specific speech rates, but that the instances of voiced and unaspirated voiceless stops in the dialogue, which do not vary significantly with rate variation, deterred VOT cue modulation by speech rate, and overrode potential habitual speech rate effects. Without more supporting evidence of speaker-specific habitual speech rate effects in conversational speech, it is difficult to conclude whether habitual speech rate in this context indeed influences subsequent phoneme categorization.

The findings of this study raise interesting questions as to exactly how the speaker-specific information in contextual speech may affect subsequent perception of durational contrasts. It should be noted, however, that the present results are limited to the range of rate manipulation used in this study. Future studies can test different rate manipulations and pinpoint the source of such a speech rate effect by varying the type of structures included in the exposure dialogue. The exposure conditions can vary from including the exact matches of the target (word-initial /p/ and /b/ in stressed position) or even the same lexical items, that is, /pi/ and /bi/, to including instances of similar sounds (e.g., word-initial /t/ and /d/), to including no instances of similar sounds, with similarity being defined at different levels of generality.

To conclude, the focus of this study was to determine whether a habitual speech rate effect would be found using a similar task and the same amount of rate manipulation, but a different type of contrast, namely, a consonantal contrast. Given the current methodological set-up, we did not find evidence for a speaker-specific habitual speech rate effect. Further research is required to examine if habitual speech rate effects on subsequent sound categorization are contingent upon the strength of interspeaker consistency in correlation with general speech rate and the particular durational contrasts, as well as the availability of relevant structure in the exposure dialogue.
